# The association of fish consumption with bladder cancer risk: A meta-analysis

**DOI:** 10.1186/1477-7819-9-107

**Published:** 2011-09-19

**Authors:** Zhongyi Li, Jianda Yu, Qilong Miao, Shuben Sun, Lingjun Sun, Houmen Yang, Liejun Hou

**Affiliations:** 1 Department of Urology, the Affiliated Hospital of School of Medicine of Ningbo University, Ningbo, Zhejiang, 315020, China

**Keywords:** Bladder neoplasms, Diet, Fish, Meta-analysis, Prevention

## Abstract

**Background:**

The association between fish consumption and risk of bladder cancer has not been established yet. The results from epidemiological studies are inconsistent.

**Methods:**

We conducted a meta-analysis of cohort and case-control studies on the relationship between fish intake and bladder cancer. We quantified associations with bladder cancer using meta-analysis of relative risk associated to the highest *versus *the lowest category of fish intake using random effect models. Heterogeneity among studies was examined using Q and I^2 ^statistics. Publication bias was assessed using the Begg's funnel plot.

**Results:**

Five cohort and 9 case-control studies were eligible for inclusion. The combined relative risk showed that fish consumption was negatively, but not significantly, associated with a decreased risk of bladder cancer (relative risk, 0.86; 95% confidence interval, 0.61-1.12). In subgroup analyses, there was no evidence that study design, geographical region, case sample size, or exposure assessment substantially influenced the estimate of effects.

**Conclusion:**

The overall current literature on fish consumption and the risk of bladder cancer suggested no association. Because of the limited number of studies, further well-designed prospective studies are needed to explore the effect of fish on bladder cancer.

## 1. Background

Bladder cancer is the second most common urologic malignancy and the seventh most common cancer in men. It has been estimated that 386,300 patients are newly diagnosed with bladder cancer worldwide in 2008, and approximately 150,200 patients were expected to die of it [1]. Depending on its stage and grade, bladder cancer may be treated with surgery, radiation therapy, chemotherapy, or immunotherapy. Because bladder cancer has the highest lifetime treatment cost of any cancer, and direct exposure to carcinogens is implicated in bladder cancer development and many potentially protective compounds are concentrated in urine, making it an ideal target for preventive therapies [2].

Smoking, occupational exposure, and chronic infections with schistosoma are the most established risk factors for bladder cancer. At present, evidence on dietary factors is also accumulating. Fish plays an important role in the usual diet worldwide and is an ideal source of n-3 polyunsaturated fatty acids, which may lower cancer risk by suppressing mutations, inhibiting cellular proliferation, and inducing cell apoptosis [3-5]. A report by the World Cancer Research Fund and the American Institute for Cancer Research on the relationship between diet and cancer concluded, based on a comprehensive review of epidemiologic studies, that fish consumption may possibly protect against cancers of the colon, rectum, and ovary [6]. Less attention, however, has been paid to the role of fish consumption on bladder cancer risk. Several epidemiological studies have examined the association between fish intake and the risk of bladder cancer; the majority of results are null, which could possibly be attributable to lack of statistical power in individual studies. Thus, we conducted a meta-analysis of all published studies to evaluate the relationship between fish consumption and bladder cancer.

## 2. Methods and materials

### 2.1 Search strategy

We identified studies by a literature search of the PubMed databases up to January 2011 with the following key words: "fish," "meat," or "diet" combined with "bladder cancer," "urothelial cancer," or "urinary tract cancer." In addition, we reviewed the reference lists from all relevant articles to identify additional studies. All searches were conducted independently by two authors. The results were compared, and any questions or discrepancies were resolved through iteration and consensus.

### 2.2 Study selection

Following criteria were used to identify relevant studies for the meta-analysis. First, they had to be case-control or cohort studies in English language. Second, the studies needed to examine fish consumption as a risk factor for bladder cancer. Last, each study should provide risk estimate together with its corresponding 95% confidence interval (CI) adjusted for at least age, sex and smoking (or sufficient information to calculate it). We also included the articles evaluating the risk of urinary tract cancer with fish consumption, for bladder cancer accounts for the overwhelming majority of tumors, and the renal pelvis and ureter are covered by the same urothelium. The term bladder cancer was used as a synonym for these neoplasms.

**Figure 1:**
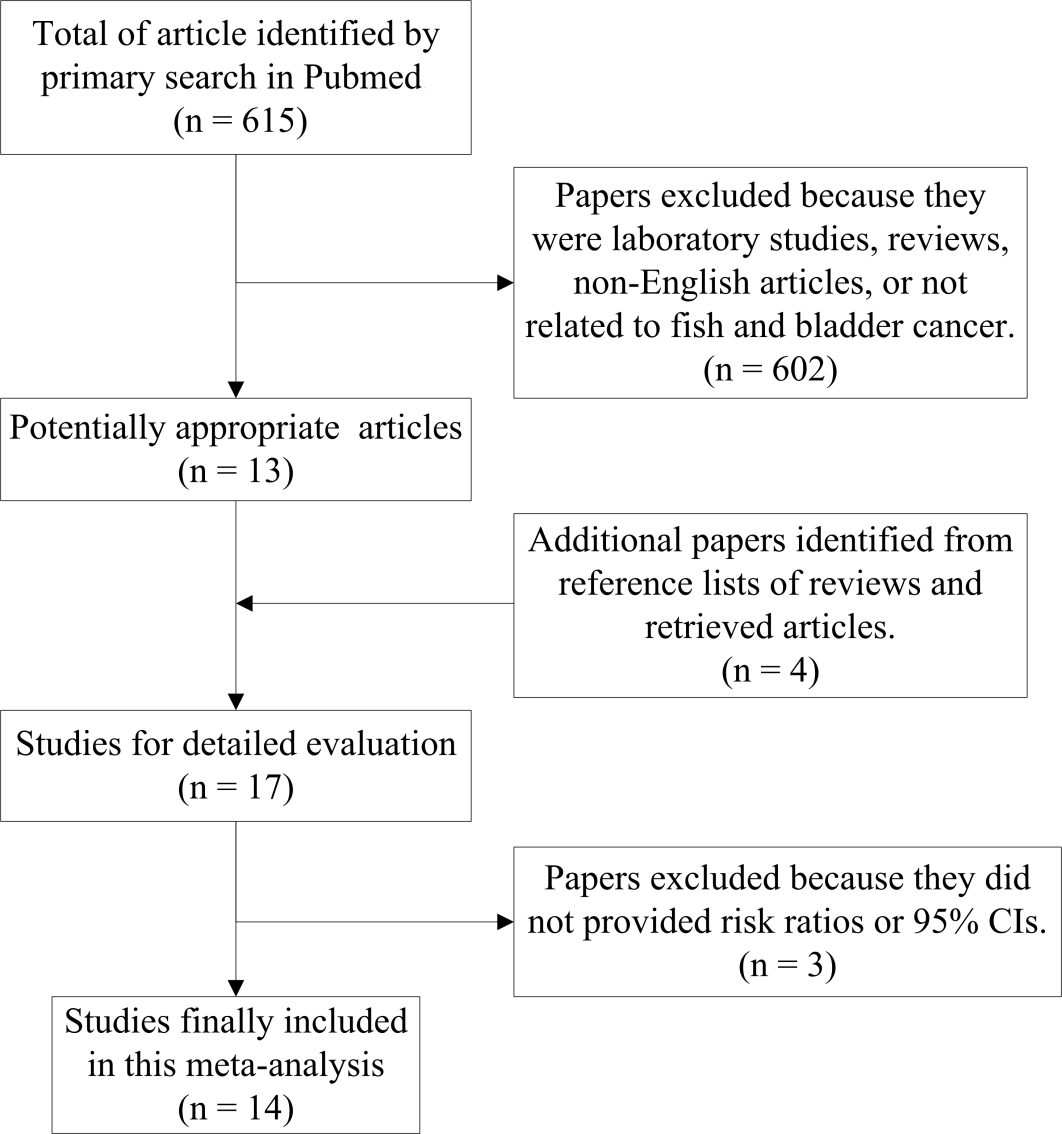
**Process of study selection for fish consumption and risk of bladder cancer**.

The process of study selection was shown in Figure 1. Seventeen potentially relevant studies were identified by searching PubMed and references of retrieved articles or reviews [3,7-22]. Three studies were excluded because one reported only odds ratio (OR) but no 95% CI [8], and two presented the ORs for meat and fish consumption together [11,13]. Thus, a total of 14 studies were included in this meta-analysis.

### 2.3 Data extraction

The following data were extracted independently by two authors from each study: the name of the first author, year of publication, the country in which the study was conducted, study design, study period, sample size, exposure of fish consumption, risk estimates with corresponding 95% CIs for highest *vs *lowest level of fish consumption, covariates controlled for in the analysis and exposure assessment. Because bladder cancer is a rare disease, OR can be interpreted as RR. For simplicity, we report all results as RR.

### 2.4 Statistical analysis

We estimated a pooled RR with 95% CI based on random-effects models, which incorporates both within and between-study variability [23]. One study reported sex-stratified RRs, we calculated the overall sex-adjusted RR by combining the two estimates with the method of Mantel and Haenszel [24]. Heterogeneity was assessed using Q-test [23] and I^2 ^score [25], and statistical significance was considered while P < 0.05. A sensitivity analysis was conducted to test the impact of each study on the pooled estimates by removing each study from the meta-analysis separately. Publication bias was assessed through visual inspection of funnel plots, and tests of Begg [26]. We performed meta-regression analysis to explore the influence of study design, geographical region and publication years in the heterogeneity. All statistical analyses were conducted using Stata (StataCorp, College Station, Texas).

## 3. Results

Table 1 presents the basic characteristics of each study included in our meta-analysis. There were 5 cohorts and 9 case-control studies (3 population-based and 6 hospital-based case-control studies). Seven studies were conducted in Europe, 3 in US/Canada, 3 in Japan, and the remaining one in Uruguay. Most studies have reported non-significant associations, and the risks were significantly decreased in 2 studies.

**Table 1:** Study characteristics of published cohort and case-control studies on fish intake and bladder cancer

Authors and publication year	Study design	Country	Study period	Cases/subjects	Fish consumption	RR (95% CI)	Variables of adjustment	Assessment
Steineck et al. 1988	Cohort	Sweden	1968-1982	80/16477	Ever vs ever	1.3 (0.8-2.2)	Age, sex and smoking	Questionnaire
Steineck et al. 1990	PCC	Sweden	1985-1987	326/719	Weekly vs more seldom	1.1 (0.7-1.8)	Age, sex and smoking	Questionnaire
Riboli et al. 1991	HCC	Spain	1985-1986	432/1221	The highest vs the first quartile	1.26 (0.86-1.84)	Age, smoking and total calories	Interview
Chyou et al. 1993	Cohort	USA	1965-1991	96/7090	≥ 5 times/wk vs ≤ once/wk	0.67 (0.26-1.67)	Age, smoking	Both techniques
Fernandez et al. 1999	HCC	Italy	1983-1996	431/7990	≥ 2 servings/wk vs < 1 serving/wk	1.4 (1.0-1.8)	Age, sex, area of residence, education, smoking, alcohol consumption, and body mass index	Interview
Nagano et al. 2000	Cohort	Japan	1979-1993	114/38540	≥ 5 times/wk vs ≤ once/wk	1.31 (0.75-2.25)	Age, gender, radiation dose, smoking status, education level, body mass index and calendar time	Questionnaire
Wakai et al. 2000	HCC	Japan	1996-1999	297/592	The highest vs the first quartile	0.86 (0.54-1.38)	Age, sex, smoking and occupational history as a cook.	Interview
Balbi et al 2001	HCC	Uruguay	1998-1999	144/720	The highest vs the first tertile	0.82 (0.49-1.36)	Age, sex, residence, urban/rural status, education, body mass index, tobacco smoking, 'mate' drinking, and total calories.	Interview
Sakauchi et al. 2005	Cohort	Japan	1988-1997	115/65184	Almost every day vs 1-2 times/month	0.36 (0.18-0.72)	Age, sex and smoking	Questionnaire
Baena et al. 2006	HCC	Spain	Not mentioned	74/163	≥ 3 times/wk vs never	0.13 (0.05-0.33)	Age, smoking, water intake	Interview
Holick et al. 2006	Cohort	US	1986-2002	736/173229	≥ 1 serving/day vs 1-3 serving/month	Men 0.71 (0.48-1.04) Women 1.33 (0.74-2.40)	Age, sex, total caloric intake, pack-years of cigarette smoking, and current smoking	Questionnaire
Garcı'a-Closas et al. 2007	HCC	Spain	1998-2001	873/1785	The highest vs the first quintile	0.9 (0.6-1.2)	Age, gender, region, smoking status, duration of smoking and quintiles of fruit and vegetable intake	Interview
Hu et al. 2008	PCC	Canada	1994-1997	1029/6068	The highest vs the first tertile	0.8 (0.6-1.1)	Age, province, education, body mass index, sex, alcohol, smoking, total of vegetable and fruit intake, and total energy intake	Questionnaire
Brinkman et al. 2011	PCC	Belgium	1999-2004	200/486	The highest vs the first tertile	0.77 (0.47-1.27)	Sex, age, smoking status, number of cigarettes smoked per day, number of years smoking, occupational exposure to PAHs or aromatic amines and energy intake.	Questionnaire

PCC: population-base case-control study, HCC: hospital-base case-control study.

**Figure 2:**
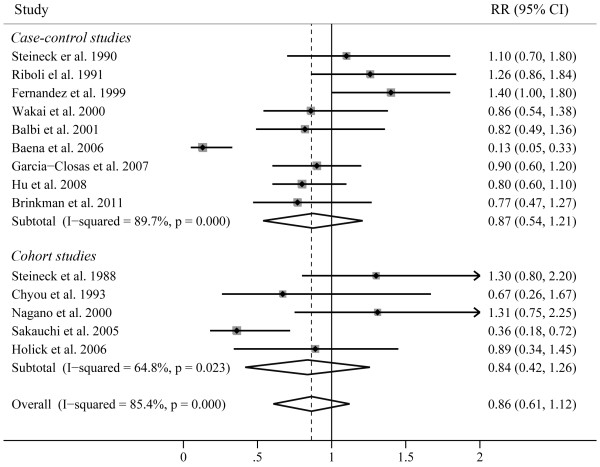
**Forest plots showing risk estimates from case-control and cohort studies estimating the association between fish consumption and risk of bladder cancer**.

**Figure 3:**
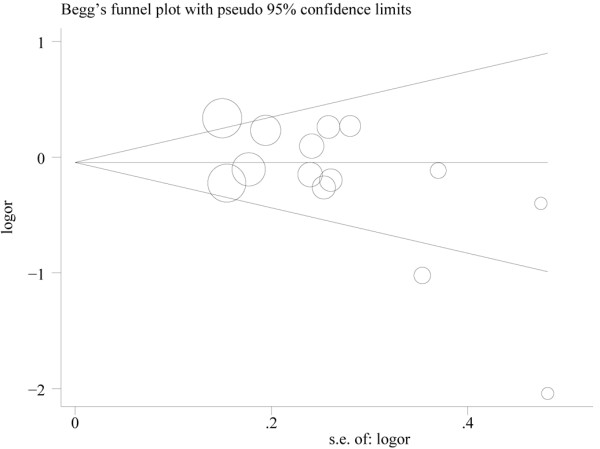
**Funnel plot of fish consumption and bladder cancer risk**.

Risk estimates for highest *vs *lowest level of fish consumption are shown in Figure 2. The summary RR of all studies, using a random effects model, did not show that fish consumption was significantly associated with decreased risk of bladder cancer (RR, 0.86; 95% CI, 0.61-1.12). There was statistically significant heterogeneity across the studies (P < 0.001, I^2 ^= 85.4%). Begg's funnel plot indicated that there was no significant publication bias (Figure 3, P = 0.101). A sensitivity analysis in which one study at a time removed was performed to evaluate the stability of the results. The summary RR ranged from 0.83 (95% CI, 0.57-1.10) (when excluding the study by Riboli et al [10]) to 0.91 (95% CI, 0.63-1.20) (when excluding the study by sakauchi et al [17]), indicating the stability of results.

To explore the source of heterogeneity, we next pooled the RR estimates by study design, geographical region, and exposure assessment (Table 2). The summary RRs neither from cohort studies (RR, 0.84; 95% CI, 0.42-1.26) nor from all case-control studies (RR, 0.87; 95% CI, 0.54-1.21) showed that fish intake was related to decreased bladder cancer risk. When we separated the population-based case-control studies from their hospital-based case-control studies, we found no apparent difference between hospital-based case-control studies (RR, 0.88; 95% CI, 0.40-1.36) and population-based case-control studies (RR, 0.83; 95% CI, 0.63-1.03). In addition, the RR estimates showed fish consumption was consistently associated with a decreased but non-significant risk of bladder cancer when separately analyzed by geographical region and exposure assessment. We also performed subgroup analysis by case sample size. The studies which included more than 200 cases of bladder cancer were defined as "large", and those with less than 200 cases were considered as "small". We found a marginally decreased risk of bladder cancer for studies of small case sample size (RR, 0.68; 95% CI, 0.34-1.02). However, the combined RR for studies with larger case sample size, which provide more reliable results, supported that fish intake was not related to risk of bladder cancer.

**Table 2:** Summary of pooled risk ratios of bladder cancer for fish consumer by study design, geographical region, and exposure assessment

Subgroup	Number of studies	Pooled RR (95% CI)	Q-test for heterogeneity	
			P value	*I^2 ^*score
Study design				
Cohort studies	5	0.84 (0.42, 1.26)	0.023	64.8%
Case-control studies	9	0.87 (0.54, 1.21)	< 0.001	89.7%
Hospital-based case-control studies	6	0.88 (0.40, 1.36)	< 0.001	92.5%
Population-based case-control studies	3	0.83 (0.63, 1.03)	0.586	0
Geographical region				
Europe	7	0.95 (0.49, 1.42)	< 0.001	91.9%
US/Canada	3	0.80 (0.58, 1.02)	0.891	0
Japan	3	0.76 (0.25, 1.27)	0.019	74.7%
Exposure assessment				
Interview	6	0.88 (0.40, 1.36)	< 0.001	92.5%
Mailed questionnaire	7	0.84 (0.58, 1.09)	0.025	58.5%
Case sample size				
Large	7	0.99 (0.82, 1.17)	0.215	28.0%
Small	7	0.68 (0.34, 1.02)	< 0.001	81.4%

Meta-regression analysis was used to explore the influence of publication year, geographical region, study design, case sample size and exposure assessment in the heterogeneity. However, none of them was identified as a possible source of heterogeneity among all the included studies.

## 4. Discussion

Diet is considered to play a very important role in preventing cancer. Fish is an important aspect of diet that has been linked favorably or unfavorably to the risk of several cancers. On the one hand, there are serious concerns about mercury and other environmental impurities that accumulated in fish. On the other hand, fish is regarded as a terrific source of polyunsaturated fatty acids. This present study is the first meta-analysis summarizing the evidence to date regarding the association between fish consumption and bladder cancer risk. Overall, the summary RR for all of the studies suggested no significant association between fish consumption and the bladder cancer risk. There was a significant heterogeneity among the studies. However, the results were also non-significant when the case-control or cohort studies were evaluated individually, or in subgroup analysis by geographical regions, case sample size and exposure assessment. Moreover, we did not identify any potential sources of heterogeneity using meta-regression analysis.

We noted that the associations between fish intake and bladder cancer risk were negative in all studies published after 2000, while the RRs for studies before 2000 tend to be positive (Table 1). This might be explained in part by the improvement of the adjustment for smoking in recent years. Cigarette smoking is one of the most important risk factors for bladder cancer, so it is possible that smoking may confound the fish-bladder cancer association if not properly controlled for, which may be particularly true in older studies, leading to spurious positive associations between fish intake and bladder cancer risk. However, we could not provide the separate meta-analyses for nonsmokers and smokers because of the few studies available.

The association between fish intake and bladder cancer is biologically plausible. Fish and fish oil are a rich source of long-chain, n-3 polyunsaturated fatty acids (PUFA), eicosapentaenoic acid (EPA) and docosahexaenoic acid (DHA). The n-3 fatty acids is suggested to reduce cancer risk via several potential mechanisms, including modulation of eicosanoid production and inflammation, angiogenesis, proliferation, susceptibility for apoptosis, and estrogen signaling, which are variables that are key drivers in cancer progression [27]. Using animal models, researchers have found that supplementing the diet of tumor-bearing mice or rats with purified n-3 fatty acids has slowed the growth of various types of cancers [28-30]. In addition, intake of oils containing EPA or DHA has also been shown to suppress cancer growth in animal studies [31-33]. However, no data regarding to the effects of fish ingredients on bladder cancer has been published.

The present study has important limitations which should be considered when interpreting our results. First, fish consumption includes fatty fish, which are much higher in the fatty acids, as well as fish that are lower in marine fatty acids, and many studies have also reported increased cancer risks associated with consumption of salted fish. However, we only assessed total fish consumption because most of these studies were not primarily designed to investigate the effect of fish consumption on bladder cancer risk, and did not specify what type of fish was consumed, providing one explanation for the heterogeneity of the study. Second, only articles published in the English language were included, and we did not search for unpublished studies or original data, although no publication bias was indicated visually or in formal statistical testing. Third, the classification of exposure varied considerably across the included studies, and the different amount of fish consumption may contribute to the heterogeneity among studies in the analysis of the highest versus the lowest intake categories.

## Conslusion

In conclusion, in this meta-analysis of 5 cohorts and 9 case-control studies, we did not found fish consumption was associated with reduced risk of bladder cancer. Given the small number of cohort studies included in this meta-analysis, further prospective cohort studies with larger sample size, well-controlled confounding factors, and more accurate assessment of fish consumption are needed to affirm the effect of fish on bladder cancer.

## Competing interests

The authors declare that they have no competing interests.

## Authors' contributions

ZL conceived of the study concept and participated in its design, data extraction, statistical analysis, manuscript drafting and editing. JY and QM participated in the literature research, manuscript drafting and editing. SS participated in design and data extraction. LS and HY participated in manuscript drafting, editing and statistical analysis. LH conceived of the study concept and participated in data analysis. All authors read and approved the final manuscript.
